# Postpartum Depression and Associated Factors Among Mothers Who Visited for Postpartum Follow‐Up in Selected Public Health Centers in Addis Ababa, Ethiopia: A Multicenter Cross‐Sectional Study Design

**DOI:** 10.1002/hsr2.72320

**Published:** 2026-04-11

**Authors:** Digafe Tsegaye Nigatu, Roman Negawo Desta, Aida Zerihun Mekonnen

**Affiliations:** ^1^ Public Health Department Yekatit 12 Hospital Medical College Addis Ababa Ethiopia; ^2^ Black Lion Specialized Hospital Addis Ababa University Addis Ababa Ethiopia

**Keywords:** associated factors, Edinburgh postnatal depression scale, postpartum depression

## Abstract

**Background and Aim:**

Postpartum depression (PPD) is a serious health concern globally, and it can have unfavorable effects on mothers, their offspring, and their families. However, in developing countries like Ethiopia, women's psychological health remains largely overlooked. We aim to assess the prevalence of postpartum depression and associated factors among mothers who visited for postpartum follow‐up and immunization of their newborns in selected public health facilities in Addis Ababa, Ethiopia, 2024.

**Methods:**

A facility‐based, cross‐sectional study was conducted. Data were collected from 380 respondents selected with random sampling technique from October to November 2024. Data were collected using interviewer‐administered, structured questionnaire and entered into Kobo Tool box and analyzed using SPSS version 26. Descriptive analysis was used to summarize general characteristics. Bivariate and multivariable regression analyses were run to identify factors associated with postpartum depression. Odds ratio (OR) with 95% confidence intervals (CI) was computed, and a *p*‐value < 0.05 was considered statistically significant.

**Results:**

A significant proportion of mothers (36.10%; (95% CI: 32.1%–41.6%); *n* = 137) were experienced a postpartum depression. Age ≥ 35 years (AOR = 2.64; 95% CI: 1.21–5.77), being Cesarean as a mode of delivery (AOR = 3.89; 95% CI: 1.36, 11.10), current pregnancy complication (AOR = 1.03; 95% CI: 1.53, 1.99), a history of mental illness [AOR = 2.66; 95% CI: (1.06–6.63)], poor and moderate social support (AOR = 1.96; 95% CI: 1.99, 3.85), (AOR = 1.93; 95% CI: 1.02, 3.65), respectively, and Intimate partner violence (AOR = 1.12; 95% CI: 1.39, 3.25) were found to show as risk factors and statistically associated with postpartum depression.

**Conclusion:**

Postpartum depression is common among the studied mothers. Being maternal age of > 35, cesarean as a mode of delivery, current pregnancy complication, mental illness history, poor and moderate social support, and intimate partner violence were factors associated with postpartum depression. Routine screening, counseling for postpartum depression should be a part of maternal healthcare services. Enhancing social and emotional support frameworks is crucial.

**Abbreviations**
DSM: diagnostic and statistical manual
EPDS: Edinburgh postnatal depression scale
PPD: postpartum depression
SDGs: sustainable development goals
Y12HMC: Yekatit 12 Hospital Medical College

## 1 Introduction

Postpartum depression is defined by the Diagnostic and Statistical Manual of Mental Disorders (DSM) as a type of depression that occurs within 4 weeks following childbirth [1]. On the other hand, the ICD‐10 describes it as the onset of depressive symptoms in the first 6 weeks postpartum [2, 3]. Further, an international expert panel recommends 3 months as the time frame for specifying postpartum onset [4]. After giving birth, a mother may undergo a range of emotions from joy and excitement to feelings of sadness and thoughts of self‐harm. The postpartum period is a crucial time when various psychological issues, such as postpartum depression (PPD), can emerge [5, 6]. It stands out as the most common psychological condition affecting women following childbirth [7].

While the exact causes of PPD are not definitively pinpointed, it is believed to stem from a combination of genetic, hormonal, psychological, and social factors. Notably, fluctuations in reproductive hormones like estrogen and progesterone are thought to significantly impact the neurobiology of PPD [8, 9]. Symptomatically, it is typically characterized by several somatic and emotional features that include desperation, sadness, nausea, changes in sleep and eating habits, decreased libido, crying bouts, anxiety, irritability, feelings of isolation, mental liability, thoughts of hurting oneself and/or the infant, and even suicidal thoughts [5, 8].

Social support is important to lowering the stress associated with caring for a new baby, as well as assisting individuals in better adjusting to the life changes that accompany parenthood. In particular, partners' perceived support has been related to lower levels of maternal and infant distress. Among the functional domains that make up social support are the provision of information (informational support), practical assistance (instrumental support), emotional sharing and empathy (emotional support), and affirmation or feedback (appraisal support) [10, 11, 12]. These different forms of support are essential for a child's healthy development and a smooth transition to parenthood.

Similar to the global landscape, the prevalence of PPD differs across sub‐regions of Sub‐Saharan Africa, with relatively highest prevalence in Western Africa (20.2%), followed by Eastern Africa and Southern Africa, where the corresponding figures were 18.6% and 18.3%, respectively [13]. A recent systematic review reported that the prevalence of postnatal depressive symptoms ranged from 3.8% to 69.9%, with a pooled estimate of 22.1% [14].

Recently, maternal mental health has caught the attention of local and international researchers as maternal mental health issues have been recognized as integral components of the bundle to reach the Sustainable Development Goals (SDGs) of reducing maternal mortality ratio and the under‐five mortality rate [15, 16].

The Ethiopian national mental health strategy for the period 2012/2013–2015/2016, developed by the Federal Democratic Republic of Ethiopia Ministry of Health (FMOH), indicated that mental health issue was the most leading non‐communicable disorder in terms of burden. Certainly, in an area of Ethiopia that is mainly rural, mental health disorders accounted for 11% of the overall disease burden, with schizophrenia and depression ranking among the 10 most significant health issues. The general depression rate stood at 5.0%, with over one in 10 pregnant women and one in 20 women after childbirth in Ethiopia experiencing undiagnosed depression [17, 18].

As a consequence, numerous studies have been carried out to identify risk factors that contribute to PPD across various social contexts and conditions. Thus, multiple risk factors for PPD have been identified, and they include socio‐demographic characteristics, obstetric related factors, and social and behavioral factors [6, 17]. It is with such a rationale that the present study is conceived to assess the prevalence and associated factors of postpartum depression among mothers visiting vaccination clinics in selected public health facilities in Addis Ababa, Ethiopia.

In Ethiopia, the primary focus of postpartum healthcare is on the obstetric and gynecological issues pertaining to the mother and the health of the newborn. Meanwhile, the psychological well‐being of the mother was given minimal attention, despite the fact that identifying mothers who may be at risk for postpartum depression early on can lead to prompt referral, correct diagnosis, and suitable treatment. Moreover, there is limited information regarding the prevalence of postpartum depression and its influencing factors in the area examined in this study, and earlier research often involved small sample sizes and overlooked key potential risk elements. Hence, this research was designed to assess the prevalence and factors contributing to postpartum depression in mothers who are receiving postpartum care and vaccination services at public health facilities in Addis Ababa, Ethiopia.

## 2 Methods and Materials

A facility‐based cross‐sectional study design was employed from October to November 2024 among women Attending Vaccination Clinics in Addis Ababa, Ethiopia, which is the capital of Ethiopia. The city has a high population density, with a total population of approximately 5,703,628 of which 49.9% (2,840,592) are women in childbearing period as of February 2024 [19].

The study included mothers who visited for a routine postpartum follow‐up visit and immunization of their newborns in the selected health facilities up to 6 weeks postpartum period. Women who are in acute psychiatric or other clinical distress or illness, those who experienced serious other life crisis postpartum were excluded.

The sample size was estimated using a single population proportion formula, considering into account a 95% confidence interval (with Z1‐*α*/2 = 1.96), an expected proportion of postpartum depression among women to be 37.4% [20], and 5% margin error and 10% nonresponse rate, the estimated final sample size was 398. Postpartum women were chosen at random from Addis Ababa's public hospitals for the study. First, three hospitals were chosen through simple random sampling (SRS) from the six hospitals owned by the Addis Ababa city administration. Gandhi Memorial Hospital (GMH), Tirunesh Beijing Hospital (TBH), and Abebech Gobena Maternal‐Child Hospital (AGMCH) were the chosen health care facilities. Every hospital had an equal chance of being included in the study using a random sampling.

Following the selection of the public hospitals, each hospital's sample size was calculated proportionately based on the total number of deliveries that were recorded over the course of the year prior to it. The number of participants recruited from each hospital was ensured to reflect the hospital's delivery volume because of proportionate allocation. In particular, 156 participants were recruited by AGMCH, 132 by GMH, and 110 by TBH.

A consecutive sampling approach was employed to recruit participants within each hospital. During the period of data collection, eligible postpartum women were reached in the maternity wards and postpartum care (PNC) units. All eligible women who visited the PPC unit during the postpartum time window were invited to participate in the recruitment process, which took place on all working days of the study period. The hospital delivery logbooks were used as a sampling frame to determine the proportionate allocation of participants until the hospital's allotted sample size was reached.

Women who were willing to participate in the study, able to give informed consent, and within 6 weeks of giving birth met the eligibility requirements. Women who were unable to participate due to severe medical conditions or critical illness were not allowed to participate.

The required data were collected via face‐to‐face interview using a structured questionnaire (Annex‐2) by involving four healthcare professionals that was designed in the English language, translated into a local language (i.e., Amharic), and then back to the English language for consistency. The Amharic version of the Edinburgh Postpartum Depression Scale (EPDS) has been validated from earlier research literature [21]. To validate the data collection tool, a 5% (*n* = 23) of the sample size was conducted before full‐scale data collection. Extensive 1 day training was provided to the data collectors. A semi‐structured interviewer‐administered questionnaire was adopted from previously published similar literatures [10, 22, 23]. The contents of the questionnaire have five parts which are the socio‐demographic, obstetric, behavioral, social support factors, and EPDS scale.

The measurement of the outcome variable for postpartum depression (PPD) was assessed using the Edinburgh Postpartum Depression Scale (EPDS). The Edinburgh Postpartum Depression Scale (EPDS) is an instrument developed to support in the identification of suspected postpartum depression symptoms. EPDS score between 1 and 9 shows the existence of certain distress symptoms that may be short lived and are less likely to impair daily capacity to perform within the home or at workplace. EPDS scores of 10–12 indicate a possible depression, which is relatively high likelihood of depression; whereas EPDS scores of 13 and higher indicate probable depression. PPD was classified into two groups: depressed, which included women who received scores of 10 or higher, and non‐depressed, which encompassed women who obtained scores lower than 10 [21]. It is the most commonly used depression screening tool in perinatal care [24, 25].

Moreover, the 14‐point Oslo Social Support Scale (OSSS‐3), a well‐known and validated short tool for measuring perceived social support in people, was used to measure the participants' level of social support. Three items make up the OSSS‐3, which measures various aspects of social support [1]: the number of close confidants or people the respondent can rely on [2]; the degree of interest and concern displayed by others; and [3] the ease of getting useful assistance from neighbors. Higher scores indicate greater perceived social support. Each item is scored on a predetermined ordinal scale, and the sum of the three item scores yields a total score ranging from 3 to 14.

Based on predetermined cutoffs, social support was analyzed as a predictor variable and divided into three levels: 3–8 points for poor social support, 9–11 points for moderate social support, and 12–14 points for strong social support. The relationship between social support and the desired outcome was investigated using these categories. Cronbach's alpha was used to assess the scale's internal consistency in the current study population. The result was a value of 0.60, which is reasonable for a short three‐item scale.

In this study history of mental illness was operationally defined as any prior diagnosis of a psychiatric disorder, such as depression, anxiety, bipolar disorder, or schizophrenia, prior to the current postpartum period. A structured questionnaire administered by an interviewer was used to collect data, which was then, when feasible, cross‐checked with medical records. For analysis, the variable was coded as either “*No*” (no history) or “*Yes*” (history of mental illness).

Any previous pregnancy‐related complication (such as preeclampsia, preterm birth, miscarriage, or stillbirth) that occurred prior to the current postpartum period is defined in this study. It is evaluated using a structured interviewer‐administered questionnaire and medical record review, and it is coded as (*No* = no history of complication, *Yes* = history of complication).

All important variables, including the outcome and major predictor variables, had their missing data evaluated. SPSS version 26 was used for analysis after Kobo Toolbox was used for data entry, coding, and cleaning. To find missing values and outliers during data cleaning, sorting, listing, frequency distributions, and cross‐tabulations were carried out. It was discovered that there was very little missing data in all of the important variables. As a result, only participants with complete information were included in the final analysis using a complete case analysis approach. To make sure that missingness was not consistently associated with participant characteristics; patterns of missing data were also investigated. No data imputation was done since there was very little missing data and no discernible pattern. Furthermore, the results were examined for stability, and because there were few missing cases, no significant variations were found, suggesting that the conclusions were solid. The general characteristics of the study participants were summarized up using descriptive statistical measures.

Bivariable and multivariable logistic regression analyses were used to examine the relationship between independent variables and postpartum depression. The crude odds ratio (COR) with a 95% confidence interval (CI) was first computed using bivariable logistic regression to ascertain the crude association between each independent variable and the outcome variable. Candidates for the multivariable logistic regression model were variables in the bivariable analysis that had a *p*‐value less than 0.25. To take into consideration for potential confounding factors and find independent predictors of postpartum depression in the multivariable analysis, adjusted odds ratios (AORs) with 95% CI were calculated. Variables with a *p*‐value < 0.05 were considered statistically significant. Based on data from relevant research and statistical significance in the bivariable analysis, potential confounders were selected. The Hosmer–Lemeshow goodness‐of‐fit test was used to evaluate the model's fitness. To make sure there was no significant multicollinearity, the Variance Inflation Factor (VIF) was also used to assess multicollinearity among independent variables. In order to guarantee the stability and dependability of the logistic regression estimates, the data were also examined for sparse cells and small frequencies using cross‐tabulations.

## Ethics Approval and Consent to Participate

This study was carried out strictly in compliance with the Declaration of Helsinki's ethical guidelines for research involving human subjects. The Yekatit 12 Hospital Medical College Institutional Review Board (IRB) provided ethical approval for all participating hospital facilities in this study (*Ref. No: Y12HMC/421/10/2024*). All eligible postpartum women were given a thorough explanation of the study's goals, methods, possible risks, and advantages prior to participation, and written informed consent (Annex‐1) was then acquired. The freedom to decline participation or to leave the study at any time without facing any repercussions was made clear to the participants. Trained research assistants who were given specialized training on participant confidentiality collected the data. All information gathered was de‐identified, safely kept in password‐protected databases, and only authorized research staff had access.

Protocols were developed to guarantee participant safety because postpartum women were using a depression screening tool. Women who showed signs of self‐harm risk or had high depression scores were immediately referred to the hospital's mental health services for assessment and assistance. Participants also received information about resources for support and counseling. To ensure clarity, comprehension, and relevance, instruments that needed translation were translated into the local language using a forward–backward translation process, evaluated by bilingual experts, culturally adapted, and pilot‐tested with a small group of postpartum women.

## 3 Results

### 3.1 Demographic Characteristics

Out of the total administered questionnaires (*n* = 398), 380 (95.5%) of the participants were consented to participate in the interview. The age distribution of participants was skewed, with a median age of 30 years and an interquartile range of 26–35. The majority 226, (59.5%) were aged 25–34 years at the time of data collection. More than one‐third 138, (36.3%) had attained up to secondary education. About half 181, (47.6%) were unemployed. Most respondents 307, (80.8%) reported no work‐family conflict. Three hundred five participants (80.3%) reported experiencing some form of marital satisfaction (Table 1).

**TABLE 1:** Demographic characteristics of respondents who visited for postpartum follow‐up in Selected Public Health Centers in Addis Ababa, Ethiopia, 2024.

Variable	Frequency (*n* = 380)	Percent (%)
Age category		
< 25 years	62	16.3
25–34 years	226	59.5
> 35 years	92	24.2
Educational level		
No formal education	84	22.1
Primary education	67	17.6
Secondary education	138	36.3
Tertiary education	91	23.9
Occupational category		
Self‐employed	82	21.6
Employed	117	30.8
Unemployed	181	47.6
Work‐family conflict		
Yes	73	19.2
No	307	80.8
Marital satisfaction		
Yes	305	80.3
No	75	19.7

### 3.2 Obstetric Characteristics of the Respondents

In this study, nearly two‐thirds of the respondents (68.1%) were reported that their pregnancies with index baby were wanted and planned. Fifty‐one of the sample population (13.4%) had a history of adverse obstetric outcomes. The majority of the mothers (79.7%) had low multiparous, reporting one to three prior births. Furthermore, a significant portion of the index babies (58.2%) were born from term pregnancies, while about one‐third was preterm (31.6%). Nearly two‐fifths of the mothers (39.2%) were delivered via natural vaginal delivery (Table 2).

**TABLE 2:** Obstetric characteristics of women who visited for postpartum follow‐up in Selected Public Health Centers in Addis Ababa, Ethiopia, 2024.

Variable	Frequency (*n* = 380)	Percent (%)
Pregnancy intention		
Wanted and planned	261	68.1
Wanted, but unplanned	65	17.1
Unwanted and unplanned	54	14.2
Bad obstetric history		
Yes	51	13.4
No	329	86.6
Parity		
Primiparous	40	10.5
Low multiparous	303	79.7
Grand multiparous	37	9.7
Gestational age		
Preterm	120	31.6
Term	221	58.2
Post‐term	39	10.3
Mode of delivery		
Natural vaginal	149	39.2
Instrumental	107	28.2
Cesarean section (CS)	124	32.6

### 3.3 Mental Health Characteristics of the Respondents

A total of 71 women (18.7%) reported having a coexisting chronic medical condition. Thirty mothers (7.9%) had previously been treated for a mental illness. Twenty‐three mothers (6.1%) admitted to using substances during pregnancy. Thirty‐seven mothers (9.7%) stated that they had experienced child death. Finally, 26 mothers (6.8%) reported having sustained some form of intimate partner violence during their last pregnancy. Slightly more than half of the infants (52.6%) were male by sex. About a quarter of the mothers (23.2%) described the index baby as not being their preferred sex (Table 3).

**TABLE 3:** Mental health related characteristics of the respondents who visited for postpartum follow‐up in Selected Public Health Centers in Addis Ababa, Ethiopia, 2024.

Variable	Frequency (*n* = 380)	Percent (%)
Comorbidity		
Yes	71	18.7
No	309	81.3
History of mental illness		
Yes	30	7.9
No	350	92.1
History of substance use		
Yes	23	6.1
No	357	93.9
Experience of child death		
Yes	37	9.7
No	343	90.3
Intimate partner violence		
Yes	26	6.8
No	354	93.2
Sex of the infant		
Male	200	52.6
Female	180	47.4

### 3.4 Social Support Profile of the Respondents

**FIGURE 1:**
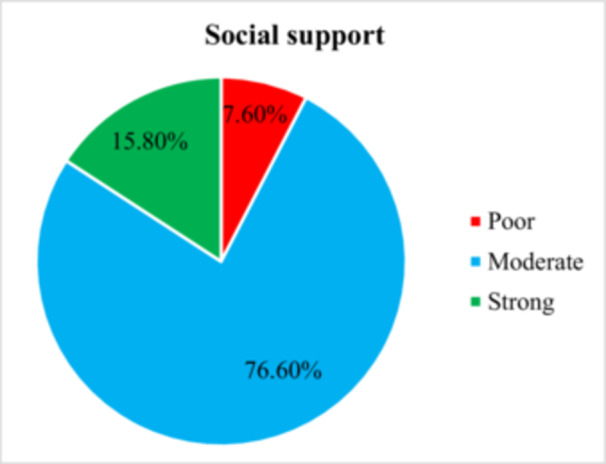
Social support profile of the respondents who visited for postpartum follow‐up in Selected Public Health Centers in Addis Ababa, Ethiopia, 2024.

The social support level was evaluated through the Oslo‐3 social support scale. Among all mothers taking part in this study, 29 (7.6%) had poor social support. The remaining 76.6% (*n* = 291) and 15.8% (*n* = 60) were shown to have moderate and strong social support, respectively (Figure 1).

### 3.5 Postpartum Depression

**FIGURE 2:**
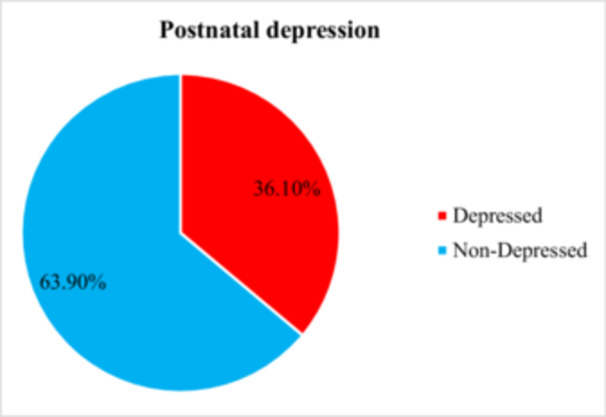
Magnitude of postpartum depression of the respondents who visited for postpartum follow‐up in Selected Public Health Centers in Addis Ababa, Ethiopia, 2024.

The study found that 137 out of 380 respondents, representing 36.1% (95% CI: 32.1%–41.6%) showed sign of postpartum depression (Figure 2).

### 3.6 Factors Associated With Postnatal Depression

In this study, 19 potential factors were considered in the logistic regression analysis. Accordingly, in bivariate logistic regression analysis 10 [10] factors (age, marital satisfaction, bad obstetric history, mode of delivery, birth of preferred sex, comorbidity, history of mental illness, experience of child death, social support, and intimate partner violence) were demonstrated an association with an outcome variable.

After adjustment for the confounding variables, four variables; (age, presence of a complication in the current pregnancy, being Cesarean as a mode of delivery, history of mental illness, intimate partner violence, and social support) were showed statistically significant association with the postpartum depression.

The odds of developing postpartum depression among aged 35 years or older mothers were twice compared to their counterparts (AOR = 2.64; 95% CI: 1.21, 5.77). The odds of developing postpartum depression among mothers who gave birth Cesarean (CS) were 3 times compared to those who gave birth via vaginal (AOR = 3.89; 95% CI: 1.36, 11.10). The odds of the mothers with a history of mental illness were twice compared to those with no history of psychiatric disorders (AOR = 2.66; 95% CI: 1.06, 6.63) to develop postpartum depression. The odds of mothers with poor and moderate social support were more likely to experience postpartum depression compared to those with strong social support (AOR = 1.96; 95% CI: 1.99, 3.85) and (AOR = 1.93; 95% CI: 1.02, 3.65), respectively. Furthermore, postpartum depression (PPD) was more common in women who experienced intimate partner violence (IPV) than in women who did not (AOR = 1.12; 95% CI: 1.39–3.25) (Table 4).

**TABLE 4:** Bivariate and multivariate logistic regression analysis of factors associated with postpartum depression among the respondents who visited for postpartum follow‐up in Selected Public Health Centers in Addis Ababa, Ethiopia, 2024.

Variable	Postpartum depression		COR (95% CI)	AOR (95% CI)	*p*‐value
	Depressed (%)	Non‐depressed (%)			
Age category					
< 25 years	35 (25.5)	27 (11.1)	1	1	**—**
25–34 years	77 (56.2)	149 (61.3)	1.54 (0.87, 2.74)	1.67 (0.86, 3.25)	0.13
≥ 35 years	25 (18.2)	67 (27.6)	2.19 (1.11, 4.32)	2.64 (1.21, 5.77)	0.01
Marital satisfaction					
Yes	18 (13.1)	57 (23.5)	1.94 (1.09, 3.44)	1.50 (0.75, 2.99)	0.25
No	119 (86.9)	186 (76.5)	1	1	
Bad obstetric history					
Yes	31 (22.6)	20 (8.2)	1.71 (0.98, 2.99)	1.03 (1.53, 1.99)	0.04
No	106 (77.4)	223 (91.8)	1	1	
Mode of delivery					
Instrumental	6 (4.4)	143 (58.8)	1.33 (0.82, 2.14)	1.95 (0.13, 3.36)	0.05
Cesarean	38 (27.7)	69 (28.4)	3.25 (1.24, 8.50)	3.89 (1.36, 11.10)	0.01
Natural vaginal	93 (67.9)	31 (12.8)	1	1	**—**
Birth of preferred sex					
Yes	36 (26.3)	23 (9.5)	1.54 (0.90, 2.62)	1.16 (0.63, 2.11)	0.63
No	101 (73.7)	220 (90.5)	1	1	
Comorbidity					
Yes	8 (5.8)	63 (25.9)	2.24 (1.26, 3.96)	1.44 (0.75, 2.78)	0.27
No	129 (94.2)	180 (74.1)	1	1	
History of mental illness					
Yes	12 (8.8)	18 (7.4)	3.80 (1.74, 8.27)	2.66 (1.06, 6.63)	0.04
No	125 (91.2)	225 (92.6)	1	1	
Experience of child death					
Yes	30 (21.9)	7 (2.9)	4.08 (1.41, 11.86)	2.93 (0.89, 9.70)	0.08
No	107 (78.1)	236 (97.1)	1	1	
Social support					
Poor	17 (12.4)	12 (4.9)	2.62 (1.46, 4.70)	1.96 (1.99, 3.85)	0.04
Moderate	93 (67.9)	198 (81.5)	1.96 (1.08, 3.56)	1.93 (1.02, 3.65)	0.03
Strong	27 (19.7)	33 (13.6)	1	1	**—**
Intimate partner violence					
Yes	19 (13.9)	7 (2.9)	0.46 (0.19, 1.07)	1.12 (1.39, 3.25)	0.03
No	118 (86.1)	236 (97.1)	1	1	

*Note:* 1: Reference category.

## 4 Discussion

In this study, the prevalence of PPD was 36.10% (95% CI: 32.1%–41.6%). Factors, such as respondents' age, bad obstetric history, mode of delivery, history of mental illness, poor and moderate social support, and intimate partner violence, were independently associated with PPD.

In this study, 36.1% women developed depressive symptoms after childbirth. This study is nearly comparable to the study conducted at Tirunesh Beijing Hospital in Addis Ababa [26], Saudi Arabia [27], Palestine [28], and Nigeria [29]. In contrast, in this study setting the prevalence is remarkably higher compared to studies conducted in Malawi [30], Pakistan [13], and a Multinational study [22]. This difference may be partially attributed to the fact that the magnitude indicated in this research relied mainly on self‐reported data, the timing of postpartum assessments, and the assessment tools and cutoff points used [31], which have been found to yield higher prevalence estimates.

This study reveals that the odds of developing postpartum depression among mothers whose age 35 years or above were 2 times higher chance of having postpartum depression compared to their counterparts (AOR = 2.64; 95% CI: 1.21, 5.77). This finding is consistent to the study conducted by Silverman et al. [32] and Muraca et al. [33]. The higher odds of postpartum depression observed in mothers of older maternal age can be attributed to the significant hormonal variations during and after pregnancy, which can affect mood and possibly lead to increased vulnerability to depression [34]. Moreover, the pressures associated with being an older parent; such as concerns about fertility, health risks to the baby, and societal expectations can consequence in increased psychological disturbance during pregnancy, which may serve as a precursor to postpartum depression [35].

The presence of current pregnancy complication was found to show association with postpartum depression. The presence of a complication in the current pregnancy was 1.03 times more likely to have PPD than their counterparts. The result was similar with studies done at Debretabor Town, Northwest Ethiopia [36], and Northwest Ethiopia [37]. Postpartum depression (PPD) was slightly more common in women who had complications during the current pregnancy, perhaps as a result of increased physical stress, fear of unfavorable outcomes for the mother or newborn, and frequent medical interventions related to pregnancy complications. During the perinatal period, these factors may increase psychological distress and lower maternal well‐being. Furthermore, obstetric complications have been associated to an increased risk of postpartum depression symptoms, according to previous studies [38, 39].

This research discovered that mothers who choose a cesarean birth experience higher levels of postpartum depression compared to those who choose for and undergo a vaginal birth. Mode of delivery using Cesarean (CS) was demonstrated an association with postpartum depression (AOR = 3.89; 95% CI: 1.36, 11.10), with a significant proportion of women experiencing postpartum depression in the Cesarean birth 124 (32.6%) and instrumental delivery 107 (28.2%) compared to the vaginal delivery. This finding is somewhat comparable with the previous reports [23]. Although the relationship is complex and influenced by various factors, the psychological state prior to delivery could play an important role in the outcome of postpartum [40].

It has been shown that no singular cause or risk element exists for postpartum depression; justifiably, the origins of postpartum depression are varied and complex. Another predictor factor that was showed association with postpartum depression in this study was a prior history of mental illness (AOR = 2.66; 95% CI: 1.06, 6.63). This finding is in agreement with various studies conducted [33, 41, 42], which identified personal history of psychiatric illness as an independent risk factor for postpartum depression. This implies that a genetic component plays an important role, whereby inherited vulnerabilities to mental health disorders can manifest during the period of postpartum [43]. The similarity can be explained as various risk elements for depression are often found during pregnancy, labor, and after childbirth. As a result, women with a history of depression should be given particular care to help lower the chances of developing PPD.

Within the domain of social support, mothers with limited social support were at a higher risk of experiencing depression in comparison to those who enjoyed strong social support. The odds of mothers with poor and moderate social support were prone to experience postpartum depression compared to their counterparts (AOR = 1.96; 95% CI: 1.99, 3.85) and (AOR = 1.93; 95% CI: 1.02, 3.65), respectively, which is consistent with previous studies [12, 21, 44]. Additionally, this finding is also in agreement with a study done at Hiwot Fana Specialized University Hospital, Harar, East Ethiopia [45, 46]. Indeed, having poor social support is among the leading factors that negatively impact mental well‐being [47, 48]. Women who receive adequate emotional and practical support from their spouses or other significant others are less likely to experience postpartum depression. In fact, gender inequality and financial difficulties combine with cultural norms surrounding the postpartum experience, endangering mental health due to the resulting disappointed expectations and exclusion, as well as exacerbation existing issues. Cultural discord, which occurs when an individual's beliefs or behaviors conflict with dominant cultural standards, could be a significant factor contributing to postpartum distress in rural Ethiopia, where the postpartum time is deeply defined by cultural practices [11, 49, 50]. Furthermore, social support serves as a protective factor against stressful life experiences by offering resources, assistance, and resilience throughout the postpartum period.

Intimate partner violence was found to show association with postpartum depressive symptoms than those who did not expose to intimate partner violence (AOR = 1.12; 1.39, 3.25). This finding is align with a study conducted in four countries within sub‐Saharan Africa [18, 46, 51], it was found that experiencing violence from a partner was associated to an increased risk of developing symptoms of depression. The reasons for the association may include the idea that violent acts can result in mental distress; particularly when such events occur in the presence of others, such as community members, acquaintances, and family, this mental distress can lead to harmful perceptions of oneself and those around them.

It is important to recognize the intrinsic limitations of this study. Owing to the social isolation and stigma toward mental illness, participants may provide inaccurate responses. This research could be influenced by recall bias as the mothers may not accurately recall earlier situations. Additionally, as a result of the cross‐sectional nature of the study, it might not establish a casual‐effect relationship. A prospective research is essential to systematically understand the nature of these factors in postpartum depression.

Another limitation is related to the comparison of prevalence and determinants with previous studies may be restricted by variations in contextual parameters, such as EPDS cut‐off scores, timing postpartum, facility vs. community sampling, and socio‐demographic characteristics despite the fact that the reviewed literature is generally relevant.

## 5 Conclusion

In summary, more than one in three mothers in this study experienced postpartum depression symptoms, which indicates a serious mental health issue for women. Postpartum depression was significantly associated with maternal age over 35, Cesarean delivery, pregnancy complications, past mental illness, a lack of social support, and intimate partner violence. These results emphasize the importance of integrating psychosocial assessment and routine mental health screening into maternal healthcare services in clinical and public health settings. Maternal well‐being is greatly affected by social support, which includes emotional, practical, informational, and appraisal support from partners, family, peers, community members, and medical professionals. Although the OSSS‐3 offers a brief assessment of perceived support, understanding the multifaceted nature and sources of social support may help clarify how it relates to symptoms of postpartum depression.

## Author Contributions

**Digafe Tsegaye Nigatu:** conception and design of the study, data acquisition, analysis and interpretation, manuscript drafting and revision, approved final version for the publication, proposal development, data analysis and interpretation, manuscript drafting and revision, approved the final version for publication. **Roman Negawo Desta:** writing – review and editing, supervision. **Aida Zerihun Mekonnen:** writing – review and editing, supervision.

## Funding

The authors have nothing to report.

## Disclosure

The lead author Digafe Tsegaye Nigatu affirms that this manuscript is an honest, accurate, and transparent account of the study being reported; that no important aspects of the study have been omitted; and that any discrepancies from the study as planned (and, if relevant, registered) have been explained.

## Conflicts of Interest

The authors declare no conflicts of interest.

## Supporting information


**Supporting File 1:** Annex 1: The study participant agreement (consent) form.

**Supporting File 2:** Annex 2: Data Collection Tool in English version.

**Supporting File 3:** Annex_3_Data_collection_tool_Translated_to_local_language

## Data Availability

The data that support the findings of this study are available from the corresponding author upon reasonable request.
