# Supplementary material for: Postpartum Depression and Associated Factors Among Mothers Who Visited for Postpartum Follow‐Up in Selected Public Health Centers in Addis Ababa, Ethiopia: A Multicenter Cross‐Sectional Study Design
Source: Health Sci Rep. 2026 Apr 11;9(4):e72320. doi: 10.1002/hsr2.72320 (PMC13069586; doi:10.1002/hsr2.72320)
Supplement: Supplementary file 1 — Supporting File 1: Annex 1: The study participant agreement (consent) form. [file HSR2-9-e72320-s003.docx]

**Annex 1: The study participant agreement (consent) form**

**Annex 1: የስምምነት ማረጋገጫ ቅጽ በአማርኛ**

በመረጃ ቅጽ ላይ የተጻፈውን የጥናት ዓላማና መረጃ በሙሉ አንብቤአለው ወይም ተነቦልኛል፣ ተረድቼውማለሁ። እሱም ስለ ድህረ ወሊድ ድብርት እንደሆነም ተነግሮኛል:: ማንነቴን በሚስጥር ይያዝልኛል:: በፈለኩት ጊዜም መረጃ መስጠጤን ማቆም እንምችል ወይንም ምንም መረጃም ከመጀመሪያዉ ጀምሮ አላመስጠት እንደምችልም ተነግሮኛል:: በዚህም መሰረት በዚህ ጥናት ላይ መረጃ ለመስጠት በፈቃዴ እሳተፋለሁ::

የተሳታፊው ፊርማ:

የመረጃው ብሳቢው ፊርማ:

ቀን:
