# Supplementary material for: Postpartum Depression and Associated Factors Among Mothers Who Visited for Postpartum Follow‐Up in Selected Public Health Centers in Addis Ababa, Ethiopia: A Multicenter Cross‐Sectional Study Design
Source: Health Sci Rep. 2026 Apr 11;9(4):e72320. doi: 10.1002/hsr2.72320 (PMC13069586; doi:10.1002/hsr2.72320)
Supplement: Supplementary file 2 — Supporting File 2: Annex 2: Data Collection Tool in English version. [file HSR2-9-e72320-s001.docx]

**Annex 2: Data Collection Tool in English version**

This is the questionnaire that will be used for the study titled, “***Postpartum Depression and Associated Factors Among mothers who visited for postpartum follow-up in Selected Public Health Centers in Addis Ababa, Ethiopia: A Multicenter Cross-Sectional Study Design***.”

001. Data collector: code ____/___/___ Name ______________________

002. Serial number______________

003. MRN: ________________

**Please encircle, fill or tick your responses in front of the questions presented below.**

**Part I: Socio-demographic information**

| Q NO | Question item | Coding categories/alternative |
| --- | --- | --- |
| 101 | Age in years | --------------- |
| 102 | Educational status | 1. Can’t read/write 2. Can read and write 3. Primary school 4. High school 5. College diploma + |
| 103 | Job/employment status | 1. Merchant  2. Civil servant  3. Private employed  4. Housewife  5. Jobless  6. Other (specify----------------------) |
| 104 | Average household monthly income | ……………………ETB |
| 105 | Work-family conflict? | 1. Yes 2. No |
| 106 | Do you think you are satisfied with your current marital relationship? | 1. Yes 2. No |

**Part II: Obstetric, behavioural and related variables**

| Q. No | Question item | Response |
| --- | --- | --- |
| 201 | Intention of the pregnancy | 1. Wanted and planned 2. Unwanted and unplanned 3. Wanted but unplanned |
| 202 | History of miscarriage or stillbirth | 1. Yes 2. No |
| 203 | Parity | __________ |
| 204 | Gestational age at birth of the index infant | ___________ weeks |
| 205 | Mode of delivery | 1. Spontaneous 2. Instrumental 3. Caesarean delivery |
| 206 | General health status of the baby as perceived by the mother | 1. Good 2. Poor |
| 207 | Sex of the infant | 1. Male 2. Female |
| 208 | Sex of the infant desired | 1. Yes  2. No |
| 209 | History of treatment for mental illness including depression | 1. Yes 2. No |
| 210 | History of chronic medical illness | 1. Yes 2. No |
| 211 | History of any substance use during pregnancy | 1. Yes 2. No |
| 212 | Ever experienced death of baby | 1. Yes 2. No |

**Part III: Social support assessing and related items**

| Q.No | Question item | Categories/alternative |
| --- | --- | --- |
| 301 | How many people are you so close to that you can count on them if you have great personal problem? | 1. None  2. 1-2  3. 3-5  4. 5+ |
| 302 | How much interest and concern do people show in what you do? | 1. None  2. Little  3. Uncertain  4. Some  5. A lot |
| 303 | How easy is it to get practical help from others if you should need it? | 1. Very difficult  2. Difficult  3. Possible  4. Easy  5. Very easy |
| 304 | History of any form of intimate partner violence during the last pregnancy | 1. Yes  2. No |

**Part IV: THE EDINBURGH POSTNATAL DEPRESSION SCALE**

As you have recently had a baby we would like to know how you are feeling. Please tick the box of the answer which comes closest to how you have felt **IN THE PAST 7 DAYS**, not just how you feel today.

| Questions | Possible answers |
| --- | --- |
| 401. I have been able to laugh and see the funny side of things: | 🞎As much as I always could  🞎Not quite so much now  🞎Definitely not so much now  🞎Not at all |
| 402. I have looked forward with enjoyment to things: | 🞎As much as I ever did  🞎Rather less than I used to  🞎Definitely less than I used to  🞎Hardly at all |
| 403. I have blamed myself unnecessarily when things  went wrong: | 🞎Yes, most of the time  🞎Yes, some of the time  🞎Not very often  🞎No, never |
| 404. I have been anxious or worried for no good reason: | 🞎No, not at all  🞎Hardly ever  🞎Yes, sometimes  🞎Yes, very often |
| 405. I have felt scared or panicky for no very good reason: | 🞎Yes, quite a lot  🞎Yes, sometimes  🞎No, not much  🞎No, not at all |
| 406. Things have been getting on top of me: | 🞎No, I have been coping as well as ever  🞎No, most of the time I have coped quite well  🞎Yes, sometimes I haven’t been coping as well as usual  🞎Yes, most of the time I haven’t been able to cope at all |
| 407. I have been so unhappy that I have had difficulty  sleeping: | 🞎Yes, most of the time  🞎Yes, sometimes  🞎Not very often  🞎No, not at all |
| 408. I have felt sad or miserable: | 🞎Yes, most of the time 🞎Yes, quite often  🞎Not very often  🞎No, not at all |
| 409. I have been so unhappy that I have been crying: | 🞎Yes, most of the time  🞎Yes, quite often  🞎Only occasionally  🞎No, never |
| 410. The thought of harming myself has occurred to me: | 🞎Yes, quite often  🞎Sometimes  🞎Hardly ever  🞎Never |

**Thank your respondent!**

Name of data collector--------------- Name of supervisor----------------------

Sig. & date -------------- Sig. & date ---------------------
