# Supplementary material for: Postpartum Depression and Associated Factors Among Mothers Who Visited for Postpartum Follow‐Up in Selected Public Health Centers in Addis Ababa, Ethiopia: A Multicenter Cross‐Sectional Study Design
Source: Health Sci Rep. 2026 Apr 11;9(4):e72320. doi: 10.1002/hsr2.72320 (PMC13069586; doi:10.1002/hsr2.72320)
Supplement: Supplementary file 3 — Supporting File 3: Annex_3_Data_collection_tool_Translated_to_local_language [file HSR2-9-e72320-s002.docx]

**Annex 2: የአማርኛ እትም መጠይቅ**

በአዲስ አበባ ኢትዮጵያ በተመረጡ የሕብረተሰብ ጤና ጣቢያዎች በክትባት ክሊኒኮች ለሚማሩ ሴቶች የድህረ ወሊድ ጭንቀት እና አስተዋፅዖ ምክንያቶች ለጥናቱ የሚውለው መጠይቅ ነው።

1. : የመረጃ ሰብሳቢ ኮድ ስም
2. ቀን

እባከው ለተጠቀሰት ጥያቄዎች መልስውን በማክበብ፣ በመሙላት፣ ምልክት በማድረግ ያስቀምጡ::

ክፍል 1: የሶሺዮ-ሕዝብ መረጃ

| የጥያቄ ቁጥር | የጥያቄ አይነት | ምድቦች / አማራጭ |
| --- | --- | --- |
| 101 | እድሜ በአመት |  |
| 102 | የትምህርት ሁኔታ | - ማንበብ/መፃፍ ማትችል - ማንበብ/መፃፍ ምትችል - 1ኛ ደረጃ ት/ቤት - 2ኛ ደረጃ ት/ቤት - ኮሌጅና ከዚያ በላይ |
| 103 | ሥራ | 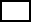 ገበሬ 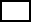 ነጋዴ 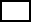 ተቀጣሪ  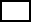 ስራ የሌለው 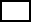 የቤት እመቤት 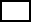 ተማሪ  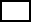 የቀን ሰራተኛ  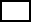 ሌላ ካለ |
| 104 | የወር ገቢ(በብር) |  |

| 105 | የስራ እና የቤተሰብ ህይወትግጭት | 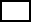 አዎ  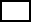 አይደለም |
| --- | --- | --- |
| 106 | ባለዎት ትዳር ደስተኛ ኖት ከሌለዎትስ ትዳር በሱ ደስተኛ ኖት? | 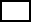 አዎ  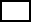 አይደለም |

ክፍል 2: የወሊድ እና ተዛማጅ ተለዋዋጮች

| የጥያቄ ቁጥር | የጥያቄ አይነት | ምላሽ |
| --- | --- | --- |
| 201 | የእርግዝናዉ ሁኔታ | 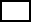 የተፈለገ እና የታቀደ  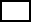 ያልተፈለገ እና ያልታቀደ  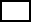 የተፈለገ ግን ያልታቀደ |
| 202 | ውርጃ ወይም ሞቶ የተወለደ ልጅ አለዎት | 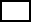 አዎ  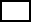 አይደለም |
| 203 | ስንት በህይወት የተወለደ ልጅ አለዎት |  |
| 204 | የአሁኑ ልጅ በስንት ሳምንቱ ነበር የተወለደው; |  |
| 205 | የወሊድ ዘዴ | 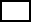 ተፈጥራዊ  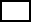 በመሳሪያ እገዛ 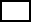 በኦፕራስዮን |
| 206 | አጠቃላይ የልጁ ጤና ሁኔታ እንደሚያዩት | 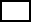 ጥሩ 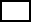 መጥፎ |
| 207 | የልጁ ፆታ | 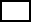 ወንድ 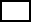 ሴት |
| 208 | የተወለደው ልጅ የሚፈልጉት ፆታ ነው | 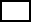 አዎ  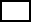አይደለም |
| 209 | ከዚህ በፊት የአእምሮ ህመም ድብርትንም ጨምሮ ገጥሞዎትያዉቃል | 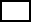 አዎ  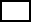 አይደለም |
| 210 | ቆየት ያለ የህክምና ህመም ታሪክ አለዎት | 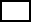 አዎ  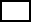 አይደለም |

| 211 | ምንም አይነት ዕፅ ሱስ አለቦት | 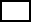 አዎ (አዎ ከሆነ ይግለጹ)  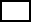 አይደለም |
| --- | --- | --- |
| 212 | ህፃን ሙቶዉበት ያውቃል | 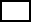 አዎ  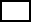 አይደለም |

ክፍል 3: የማህበራዊ ድጋፍ ግምገማ ንጥል ነገሮች

| የጥያቄ ቁጥር | የጥያቄ አይነት | ምድቦች / አማራጭ |
| --- | --- | --- |
| 301 | ትልቅ የግል ችግር ካጋጠመዎት በእነሱ ላይ እምነት የሚጥሉባቸው ስንት ሰዎች በቅርብሽ አሉ? | 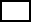 ምንም 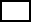 1-2  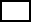 3-5  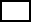 5+ |
| 302 | ሰዎች በሚያደርጉት ነገር ምን ያህል ፍላጎት እና አሳቢነት ያሳያሉ? | 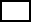 ምንም 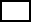 ትንሽ  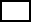 እርግጠኛ ያልሆነ 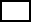 አንዳንድ  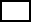 ብዙ |
| 303 | ካስፈለገዎ ተግባራዊ እርዳታ ከሌሎች ማግኘት ምን ያህል ቀላል ነው? | 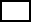 በጣም ከባድ 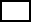 አስቸጋሪ 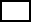 ይቻላል  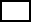 ቀላል  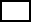 በጣም ቀላል |
| 304 | የቅርብ አጋር ምንም አይነት ጥቃት ታሪክ አሎት | 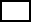 አዎ  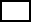 አይደለም |

ክፍል 4: የኤዲንበርግ የድህረ ወሊድ ድብርት መጠን

በቅርቡ ልጅ እንደወለዱ, ምን እንደሚሰማዎት ማወቅ እንፈልጋለን. እባኮትን የመልስ ሳጥኑ ላይ ምልክት ያድርጉ። በአለፉት 7 ቀናት ውስጥ ከተሰማዎት ስሜት ጋር ቅርብ ነው እንጂ ዛሬ ያለዎትን ስሜት ብቻ አይደለም።

| ጥያቄዎች | ሊሆኑ የሚችሉ መልሶች |
| --- | --- |
| 401. መሳቅ እችላለሁ እና የነገሮችን አስቂኝ ገጽታ ለማየት እችላለሁ | 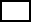 በጭራሽ  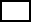 በእርግጠኝነት  አሁን በጣም ብዙ አይደለም  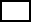 አሁን በጣም ብዙ አይደለም  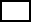 ሁልጊዜ |
| 402. ነገሮችን በደስታ በጉጉት እጠብቃለሁ | 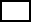 በጭራሽ |

|  | 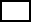 በእርግጠኝነት ከበፊቱ ያነሰ  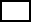 ከበፊቱ ያነሰ 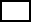 ከመቼውም ጊዜ  በላይ |
| --- | --- |
| 403. ነገሮች ሲበላሹ ራሴን ከሚገባዉ በላይ እወቅስላዉ | 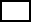 አዎ ብዙ ጊዜ  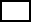 አዎ አንዳንድ ጊዜ 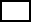 ከስንት አንዴ  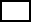 ምንም ፈጽሞ |
| 404. ያለ በቂ ምክንያት እረበሻለዉ ወይም እጨነቃለዉ | 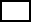 አዎ ብዙ ጊዜ 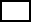 አዎ አንዳንዴ 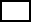 ከስንት አንዴ  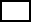 አይ በጭራሽ |
| 405. ያለ በቂ ምክንያት ፍርሃት ወይም ድንጋጤ ይሰማኛል | 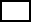 አዎ በጣም ብዙ ጊዜ  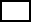 አዎ አንዳንዴ 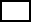 ከስንት አንዴ  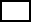 አይ በጭራሽ |
| 406. ነገሮች በላዪ ላይ ሸክም ይሆኑቢኛል/ይደራረቡብኛል | 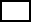 አይ፣ እኔ  እንደቀድሞው በደንብ እቋቋማለዉ  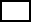 አይ ብዙ ጊዜ በደንብ እቋቋማለዉ  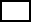 አዎ፣ አንዳንድ ጊዜ  እንደተለመደው መቋቋም  አልችልም 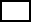 አዎ ብዙ ጊዜ  መቋቋም  አልችልም |
| 407. በጣም ደስተኛ ስላልሆንኩ መተኛት እቸገራለዉ | 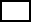 አዎ ብዙ ጊዜ 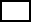 አዎ አንዳንዴ 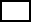 ከስንት አንዴ  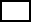 አይ በጭራሽ |
| 408. ሀዘን ወይም ተራነት ስሜት ይሰማኛል | 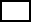 አዎ ብዙ ጊዜ |

|  | 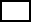 አዎ አንዳንዴ  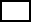 ከስንት አንዴ 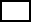 አይ በጭራሽ |
| --- | --- |
| 409. በጣም ስለሚከፋኝ አለቅሳለዉ | 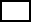 አዎ ብዙ ጊዜ 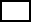 አዎ አንዳንዴ 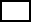 ከስንት አንዴ  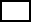 ምንም ፈጽሞ |
| 410. ራሴን የመጉዳት ሀሳብ ወደ እኔ ይመጣል | 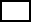 አዎ ብዙ ጊዜ 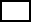 አንዳንዴ  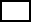 ከስንት አንዴ  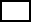 በጭራሽ |

ምላሽ ሰጪዎን እናመሰግናለን!

የመረጃ ሰብሳቢው ስም --------------- የተቆጣጣሪው ስም ------- ፊርማ ፊርማ

ቀን ቀን
